# Impact of the covid-19 pandemic on mental health and sexuality of female doctors

**DOI:** 10.1371/journal.pone.0281321

**Published:** 2023-07-10

**Authors:** Nadine de Souza Ziegler, Gabriel Cardozo Muller, Fernanda Santos Grossi, Rodolfo de Carvalho Pacagnella, Julia Schneider Hermel, Janete Vettorazzi

**Affiliations:** 1 Graduate Program in Health Sciences: Gynecology and Obstetrics, Faculty of Medicine (FAMED), Universidade Federal do Rio Grande do Sul, Porto Alegre, Rio Grande do Sul, Brazil; 2 Nucleus of Studies and Research in Sexuality of Rio Grande do Sul (NEPeSex), CNPq, Porto Alegre; 3 Graduate Program in Epidemiology, Universidade Federal do Rio Grande do Sul, Porto Alegre, Rio Grande do Sul, Brazil; 4 Department of Obstetrics and Gynecology, University of Campinas, Campinas, São Paulo, Brazil; 5 Hospital Moinhos de Vento, Porto Alegre, Rio Grande do Sul, Brazil; 6 Service of Obstetrics and Gynecology, Hospital de Clínicas de Porto Alegre, Porto Alegre, Brasil; China University of Mining and Technology, CHINA

## Abstract

**Background:**

COVID-19 pandemic has changed people’s lives around the world due to restrictive measures adopted by governments. The impact of this change on female sexuality needs to be further investigated, particularly between female doctors who are more at risk as they are directly involved with health care services.

**Methods:**

An online survey has been filled out by female doctors. The questionnaire evaluates sexual function, depression, anxiety, burnout, sociodemographic and professional data, and it was answered during the peak of COVID-19 pandemic in Brazil. The main outcome is female doctors’ sexual function during COVID-19 pandemic, which was evaluated by analyzing FSFI questionnaires. The secondary outcome is related to their mental health, assessed via depression, anxiety and burnout questionnaires.

**Results:**

A sample of 388 female doctors filled out the questionnaire. The median age was 34.0 (29.0, 43.0) years old. The total FSFI median score was 23.8 [18.9, 26.8] with desire domain median of 5.0 [3.0, 7.0]. In our sample, 231 (59.5%) women had depression and/or anxiety, out of these, 191 (82.7%) had depression and 192 (83.2%), anxiety. From these samples of doctors with depression and/or anxiety, 183 (79.2%) had sexual dysfunction.

**Conclusion:**

This finding suggests that doctors are experiencing a high risk of sexual dysfunction and mental illness during the COVID-19 outbreak. A high index of depression and/or anxiety was shown in the studied population, with almost 80% of them reaching criteria for sexual dysfunction. Working in the frontline is related to worse mental health conditions. Depression and anxiety were found as potential mediators of burnout effect on sexual function.

## Introduction

From December 2019 onwards, the world has been facing the coronavirus disease (COVID-19), and since March 2020, the World Health Organization (WHO) declared the COVID-19 outbreak as a pandemic. Clinical manifestations vary from asymptomatic infections to a severe respiratory disease [1]. The virus is transmitted from person to person mainly through respiratory droplets from a distance of up to 2 meters. Social distancing and stay-at-home measures have been recommended along with hygiene measures to prevent the spread of the virus.

As a result of this situation, healthcare professionals, who are directly involved with diagnosis, treatment and care of COVID-19 patients are at a high risk of developing psychological stress and symptoms related to mental health [2]. Women, younger and less experienced people, and particularly frontline workers who are in the risk group should be followed closely. Some data indicate that the excessive workload (increased overall number of patients cared for and increased weekly working hours, working both daytime and night shifts), reduced logistic support, decreased support from peers and supervisors and lower feeling of occupational competence during COVID-19 related tasks cause a more emotional impact in frontline physicians [3]. In Italy, a large percentage of healthcare professionals reported high scores in at least one of the Maslach Burnout Inventory domains: particularly, more than 1 out of 3 showed high score in Emotional Exhaustions and 1 out of 4 reported at high levels of Depersonalization, while only around 15% reported low levels of Personal Gratification [4]. Nursing teams have also been impacted by the pandemic, with an accentuated damage related to occupational stress, burnout syndrome, minor psychological disorders, and moral suffering [5].

COVID-19 lockdown has dramatically impacted the sexual health of the population [6]. A significant decrease in general satisfaction with their sexual life can be observed in cis women in Germany since the beginning of the COVID-19 pandemic, along with an overall decrease in the frequency of sexual contacts and masturbation [7]. In Latin America, a negative association between the impact of the pandemic and erectile and sexual function was observed, with greater implications among the individuals who live with their partners [8].

Data about how the pandemic has influenced depression, anxiety, burnout, as well as sexuality in female doctors, are rare in literature. Such pieces of information could provide highlights to improve the quality of life of these professionals.

This study aims to evaluate depression, anxiety and burnout mediating female doctors’ sexual function during the COVID-19 pandemic, and its relation to their work routine and sociodemographic data.

## Materials and methods

### Participants

This is a cross-sectional study with data collected between July 20th and August 20th, 2020, the first peak of the outbreak in Brazil. The target population of this sample is composed of female doctors who work in both public and private health systems in Brazil, including office and hospital care. The data presented in this manuscript are part of a large project that is evaluating health professionals. The participants were recruited by convenience. Professionals from two tertiary hospitals received an invitation through their institutional email to take part in the survey. Furthermore, others were recruited via digital media and social networks. The online survey was conducted using Google Forms web survey. The link to the questionnaire was distributed through social media, email, WhatsApp medical groups and sent to personal contacts of the research group members, hence, we could not assess the percentage of respondents among all invitees. In the invitation of the questionnaire, it was specified that it was a survey targeted at health professionals (doctors, residents, nurses and nurse technicians). The questionnaire was only completed when all questions were answered, thus, all participants were included in the study. In this manuscript, we present female doctor’s data. All the 388 participants in this group were included in the study.

This research was approved by the Ethics Committee of *Hospital de Clínicas de Porto Alegre* and *Hospital Moinhos de Vento*. For both hospitals, data collection started only after approval (Plataforma Brasil, CAAE 32907020.7.3001.5330, 32907020.7.0000.5327). Before beginning the survey, participants agreed with the informed consent.

### Measurements

The survey took approximately 15 minutes to complete and consisted of multiple validated questionnaires. Sociodemographic data included general demographics characteristics (age, income, educational background), relationship data and length of union, data about professional activity, for instance, changes in routine and daily working hours, health aspects, such as comorbidities, medications in use and contraception methods.

#### Sexual function

Their sexual function was assessed via *Female Sexual Function Index* (FSFI), in a validated version for Portuguese [9]. FSFI is a 19-item, self-report measure of female sexual function [10]. This tool evaluates the overall sexual function and the domains: desire, arousal, lubrication, orgasm, satisfaction and pain. A total score ≤ 26.55 is predictive of relevant sexual dysfunction [11].

#### Psychiatric illness

In this study, the participants who were considered to have a psychiatric illness were those with a positive test for depression, anxiety or burnout, by each score criteria, as described below:

*Depression.* A tool for major depressive disorder screening was used in a translated and validated version in Portuguese called Patient Health Questionnaire-9 (PHQ-9) [12]. It is a 9-item self-administered instrument used for detecting depression and assessing severity of depression. PHQ-9 items reflect the 9 symptoms of major depressive disorder [13] focused on the Diagnostic and Statistical Manual of Mental Disorders, 4th edition (DSM-IV). Each item was scored on a scale of 0–3 (0 = not at all; 1 = several days; 2 = more than a week; 3 = nearly every day) based on the last 2 weeks. The PHQ-9 total score ranges from 0 to 27 (scores of 5–9 are classified as mild depression; 10–14 as moderate depression; 15–19 as moderately severe depression; >20 as severe depression) [14]. A score of 9 was used as a cutoff for depressive disorder.

*Anxiety.* The Generalized Anxiety Disorder 7-item scale (GAD-7) is a 7-item self-report measure of generalized anxiety symptoms grouped into one factor of generalized anxiety. A translated and validated version in Portuguese was used. Respondents score each item in a 4-point scale based on how often they have been bothered by the described symptoms over the last two weeks (not at all = 0; several days = 1; more than half the days = 2; nearly every day = 3). Total scores range from 0 to 21, with higher scores reflecting higher severity levels of Generalized Anxiety Disorder (GAD) [15]. A score of 10 was used as a cutoff for generalized anxiety.

*Burnout.* Maslach Burnout Inventory—Human Services Survey (MBI-GSS) was used for burnout screening in a specific version for healthcare professionals, translated and validated version in Portuguese [16]. It is a 22-item self-reported tool that presents affirmative phrases about the feelings and attitudes of professionals regarding their work. Three dimensions of the Burnout Syndrome were evaluated in different subscales: emotional exhaustion, depersonalization and personal accomplishment. Each phrase is answered using a Likert 7-point scale (0 = never; 1 = a few times a year; 2 = monthly; 3 = a few times a month; 4 = every week; 5 = a few times a week; 6 = every day). Each dimension is evaluated with their scores added and evaluated separately [17, 18].

### Statistics

Data was processed and analyzed using R (R Core Team, 2021. R: A language and environment for statistical computing. R Foundation for Statistical Computing, Vienna, Austria) and Rstudio (v1.4.1, RStudio Team, 2021. RStudio: Integrated). Quantitative variables were analyzed in accordance with its distribution symmetry, expressed in either mean and standard deviation or median and interquartile range. In this context, either student t or Mann-Whitney test. To compare differences between groups in qualitative variables, Fisher exact test was used.

In order to better evaluate the association between psychiatric conditions and sexual dysfunction, controlling for potential confounders, we used a multivariable linear regression model with robust variances, since homoscedasticity of residuals assumption was violated, with dependent variable FSFI score. Variables were selected in accordance with a literature review added one at a time, and the best fit model was obtained by analyzing log-likelihood, Bayesian and Akaike information criteria. Variance Inflation Factors (VIF) above 5 were used as a cutoff for collinearity.

Also, before using Structural Equation Models for assessing mediation of burnout effect on sexual function by psychiatric disorders, we used Spearman correlations corrected for multiple comparisons by Holm-Bonferroni method. These correlations were displayed in heatmaps, with negative correlations expressed in blue and positive expressed in red, and intensity of color proportional to strength of correlation. Sphericity was assessed both by Kaiser–Meyer–Olkin criteria and Bartlett test, reliability by Cronbach alpha estimated through bootstrap confidence intervals, as well by autocorrelations.

To assess the relationship between burnout, depression and anxiety with sexual function, a diagonally weighted least squares (WLSMV, since most of the data was ordinal or non-normal) SEM was performed. Fit metrics were used in order to evaluate the model, with a root mean square error approximation (RMSEA) less than 0.05, comparative and Tucker fit indexes greater than 0.95 and a ratio between X^2^and degrees of freedom less than 3. Before fitting any model, an extensive review of literature was made, and burnout was used as an exogenous latent variable, and depression and anxiety as latent mediators’ variables, sexual function was used as latent dependent variable. Covariances were added among depression and anxiety latent variables. Alterations on model only were made if modification index in covariance was greater than 10, respecting theoretical rationale and maintaining model structure [19].

## Results

Descriptive data from the study population, grouped in accordance with the presence of psychiatric illness, are summarized on Table 1. The average age of the female doctors was 34.0 (IQR 29.0–43.0) years old, 235 (60%) had no children and 326 (84%) had a steady partner, with a median of relationship of 5.0 (1.0–12.0) years. 202 (52.1%) felt they worked in the frontline against COVID-19. The frequency of sexual intercourse of 1 to 2 times a month was reported by 151 (38.9%) participants; once a week by 121 (31.1%) and 2 to 3 times a week, by 89 (22.9%) participants.

**Table 1:** General characteristics. (N = 388).

	Overall	No	Yes	*p* value
n	388	157	231	
Mental illness = Yes (%)	231 (59.5)	0 (0.0)	231 (100.0)	<0.001
Age (median [IQR])	34.00 [29.00, 43.00]	38.00 [32.00, 47.00]	32.00 [29.00, 38.00]	<0.001
BMI (median [IQR])	23.05 [20.94, 25.88]	22.38 [20.52, 25.35]	23.42 [21.12, 25.95]	0.079
Relationship duration (median [IQR])	5.00 [1.00, 12.00]	7.50 [2.00, 19.25]	5.00 [1.00, 10.00]	<0.001
Steady partner = Yes (%)	326 (84.0)	141 (89.8)	185 (80.1)	0.011
Academic credentials (%)				<0.001
Medical degree	71 (18.3)	10 (6.4)	61 (26.4)	
PhD	32 (8.2)	24 (15.3)	8 (3.5)	
Specialized subjects	215 (55.4)	91 (58.0)	124 (53.7)	
Master	70 (18.0)	32 (20.4)	38 (16.5)	
Menstrual cycle (%)				0.001
Irregular during pandemic	37 (9.5)	5 (3.2)	32 (13.9)	
Same pattern	193 (49.7)	85 (54.1)	108 (46.8)	
Do not menstruate	158 (40.7)	67 (42.7)	91 (39.4)	
Physical exercise more than 150 minutes/week = Yes (%)	154 (39.7)	79 (50.3)	75 (32.5)	<0.001
Alcohol consumption (%)				0.698
Once a month	42 (10.8)	17 (10.8)	25 (10.8)	
4 or more times a week	48 (12.4)	18 (11.5)	30 (13.0)	
2 to 3 times a week	111 (28.6)	51 (32.5)	60 (26.0)	
2 to 4 times a month	136 (35.1)	53 (33.8)	83 (35.9)	
Never	51 (13.1)	18 (11.5)	33 (14.3)	
Sexual intercourse more than 4 times a week = Yes (%)	321 (82.7)	134 (85.4)	187 (81.0)	0.277
Sexual intercourse (%)				<0.001
1 to 2 times a month	94 (24.2)	38 (24.2)	56 (24.2)	
Once a month	57 (14.7)	8 (5.1)	49 (21.2)	
Once a week	121 (31.2)	51 (32.5)	70 (30.3)	
2 to 3 times a week	89 (22.9)	46 (29.3)	43 (18.6)	
More than 3 times a week	27 (7.0)	14 (8.9)	13 (5.6)	
Work in the frontline = Yes (%)	202 (52.1)	61 (38.9)	141 (61.0)	<0.001

In our sample of 388 respondents, 231 (59.5%) women had depression and/or anxiety, of these, 191 (82.7%) had depression and 192 (83.2%), anxiety. From these samples of doctors with depression and/or anxiety, 183 (79.2%) have sexual dysfunction; this population in the other group without psychiatric conditions is 99 (63.2%), (Fisher exact test, p-value = 0.001). The total scores are shown in Table 2.

**Table 2:** Total scores and by domain of the tests used to assess depression, anxiety, Burnout Syndrome and sexual function. (N = 388).

	Overall	No	Yes	*p* value
n	388	157	231	
Sexual disfunction = Yes (%)	282 (72.7)	99 (63.1)	183 (79.2)	0.001
Burnout = Yes (%)	9 (2.3)	0 (0.0)	9 (3.9)	0.013
Depression = Yes (%)	191 (49.2)	0 (0.0)	191 (82.7)	<0.001
Anxiety = Sim (%)	192 (49.5)	0 (0.0)	192 (83.1)	<0.001
GAD-7 total score (median [IQR])	9.00 [6.00, 14.00]	5.00 [3.00, 7.00]	13.00 [10.00, 16.00]	<0.001
PHQ-9 total score (median [IQR])	8.00 [5.00, 13.00]	5.00 [3.00, 7.00]	12.00 [9.00, 16.00]	<0.001
Burnout EE (median [IQR])	23.50 [15.00, 32.25]	17.00 [11.00, 22.00]	30.00 [20.00, 36.00]	<0.001
Burnout RP (median [IQR])	39.00 [35.00, 43.00]	42.00 [40.00, 45.00]	37.00 [33.00, 40.00]	<0.001
Burnout Desp (median [IQR])	6.00 [2.00, 10.00]	4.00 [1.00, 7.00]	7.00 [3.00, 12.00]	<0.001
FSFI desire domain (median [IQR])	5.00 [3.00, 7.00]	6.00 [3.00, 7.00]	4.00 [3.00, 7.00]	0.098
FSFI arousal domain (median [IQR])	4.50 [3.60, 5.40]	4.80 [4.20, 5.40]	4.20 [2.70, 5.10]	<0.001
FSFI lubrication domain (median [IQR])	5.40 [4.50, 6.00]	5.70 [4.80, 6.00]	5.10 [3.60, 6.00]	<0.001
FSFI orgasm domain (median [IQR])	4.80 [3.20, 5.60]	5.20 [4.00, 6.00]	4.40 [2.80, 5.60]	<0.001
FSFI satisfaction domain (median [IQR])	4.80 [2.80, 5.60]	5.20 [4.40, 5.60]	4.00 [2.40, 5.20]	<0.001
FSFI pain domain (median [IQR])	1.20 [1.20, 2.40]	1.20 [1.20, 1.60]	1.20 [1.20, 2.40]	0.194
FSFI total score (median [IQR])	23.80 [18.95, 26.80]	24.90 [22.50, 27.60]	22.50 [17.10, 25.90]	<0.001
Mental illness = Yes (%)	231 (59.5)	0 (0.0)	231 (100.0)	<0.001

We observed that 202 (52%) doctors declared they were working in the frontline against COVID. Noteworthy, 141 (61%) met the criteria for anxiety and/or depression (Fisher exact test, p-value<0.001).

The total FSFI median score was 23.9 [18.9, 26.8] with median desire domain of 5.0 [3.0, 7.0]. Approximately 72.2% (282) of women scored less than 26.6 in the FSFI, indicating they likely met diagnostic criteria for Female Sexual Dysfunction (FSD), in this group, 79.2% also met criteria for anxiety and/or depression (Fisher exact test, p-value = 0.001).

To better evaluate association among the presence of psychiatric diseases with FSFI score and controlling for confounding, linear regression models were made (Table 3).

**Table 3:** Linear multivariable models. Results of fitted linear models, each adjusting for additional variable, with its **β** coefficients and 95% CI (corresponding to psychiatric disease presence), statistics (t and *p* values), and model fit measures (log-likelihood, Akaike and Bayesian Information Criteria). Model 1: Unadjusted for psychiatric disease; Model 2: Adjusted for age above 40 years; Model 3: Adjusted for age above 40 years, sexual intercourse in the last four weeks; Model 4: Adjusted for age above 40 years, sexual intercourse in last four weeks, physical exercise more than 150 minutes/week. Model 5: Previous variables and sexual orientation; Model 6: Previous variables + steady partners; Model 7 (full model): previous variables + menstrual cycle.

	β	95% CI	t value	*p*-value	logLik	AIC	BIC
Model 1	-3.09	[-4.46, -1.72]	-4.4428	1.16E-05	-1305.50	2616.99	2628.88
Model 2	-3.53	[-4.88, -2.18]	-5.1309	4.58E-07	-1303.20	2614.41	2630.25
Model 3	-2.83	[-3.98, -1.68]	-4.8371	1.91E-06	-1222.21	2454.43	2474.23
Model 4	-2.61	[-3.77, -1.46]	-4.4463	1.15E-05	-1218.68	2449.36	2473.13
Model 5	-2.65	[-3.80, -1.49]	-4.5055	8.82E-06	-1215.64	2447.29	2478.98
Model 6	-2.55	[-3.70, -1.40]	-4.363	1.66E-05	-1213.68	2445.36	2481.01
Model 7	-1.47	[-3.65, -1.29]	-4.1304	4.46E-05	-1212.84	2447.67	2491.24

In all models, fulfilling criteria for a psychiatric disease presented a significant negative association with FSFI, specially on model with best fit (Unadjusted: **β** = -3.09, 95% CI [- 4.46, -1.72], Model 2: **β** = -2.47, 95% CI [-3.77, -1.46]). Noteworthy, even in full adjusted model, significance was present (**β** = -2.61, 95% CI [-3.65, -1.29]).

In model 2, age equal to or greater than 40 years (**β** = -3.53, 95% CI [-2.60, 0.09]), presented a significant negative association with FSFI total score. This association was lost in fitted model (**β** = -1.13, 95% CI [-2.46, 0.20]). In addition, in the adjusted models to practice physical exercises regularly (Model 4: **β** = 1.60, 95% CI [0.37, 2.49]) and maintaining sexual intercourse in the four previous weeks (Model 3: **β** = 10.82, 95% CI [8.48, 13.16], Model 4: **β** = 10.94, 95% CI [8.65, 13.22]) presented a positive significant impact on sexual function measured by FSFI. In all models, variance inflation factors (VIFs) were below 2, pointing to absence of collinearity.

**Fig 1:**
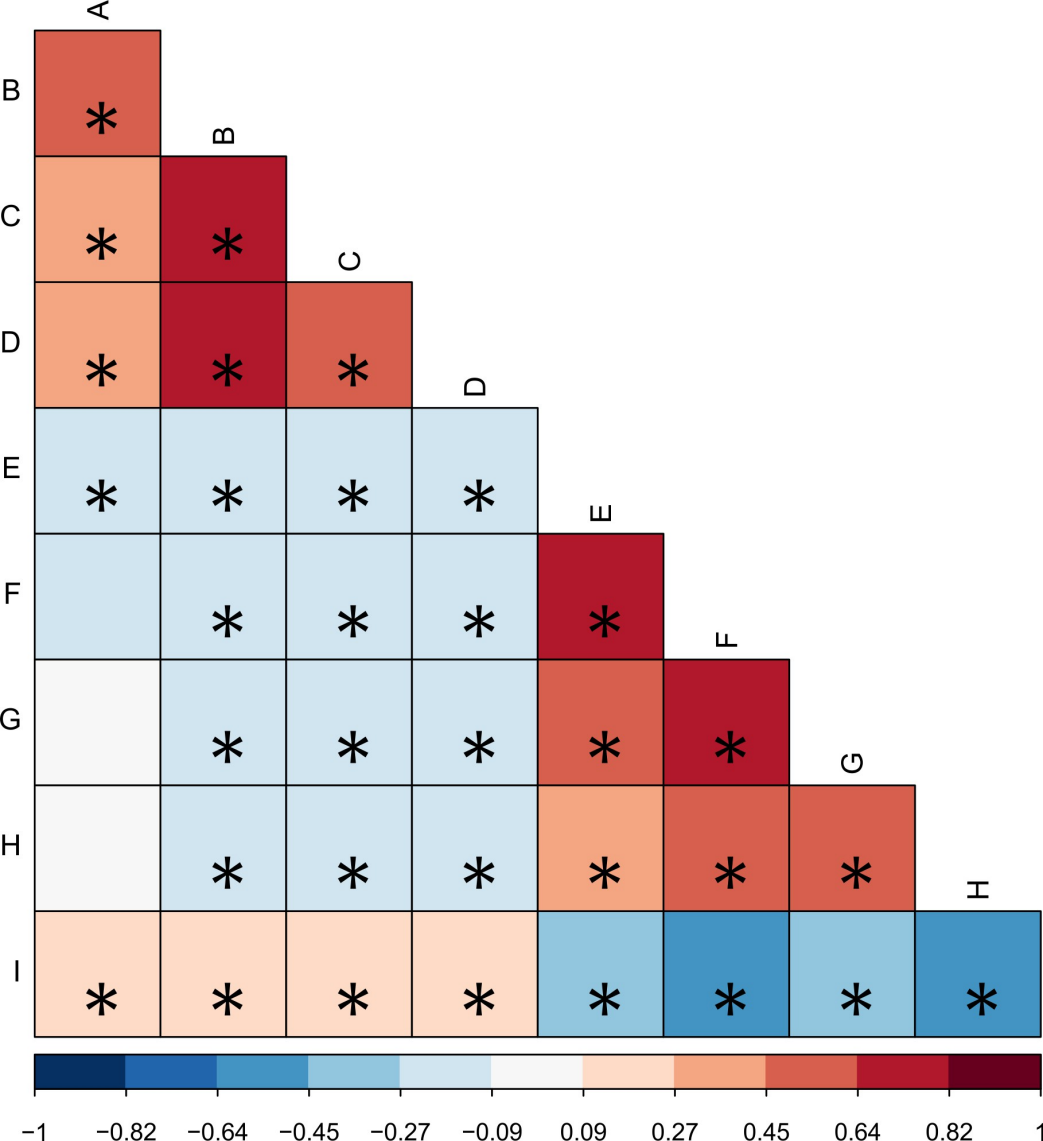
Heatmap of questionnaires. Horizontal and vertical labels represent each questionnaire score. Correlation coefficients are expressed by colors according to its color scale, represented from blue to red, meaning negative/inverse to positive/direct correlations. Statically significant correlations (p<0.05) are displayed with an asterisk on its square. Each letter represents one dominium/score: A—FSFI desire dominium, B—FSFI arousal dominium, C—FSFI lubrication dominium, D—FSFI orgasm dominium, E—GAD-7 total score, F—PHQ-9 total score, G—Burnout Maslach Emotional Exhaustion, H—Burnout Maslach Depersonalization, I—Burnout Maslach Personal Realization.

Before using structural equation models, we assessed correlations among scales through heatmap (or correlation matrix). It can be seen overall moderate and positive correlation between GAD and PHQ questions and scales. Depressive mood and tiredness presented significant moderate correlations with all GAD-7 scores (Fig 1).

Also, "difficulty to relax" (question 4—GAD) presented a strong correlation with depressive mood, tiredness, feeling guilty and worthlessness and concentration problems. The item 3 (“To be worried about many things”) presented a strong correlation with difficulty to relax.

In FSFI scores, we analyzed correlations on the total score of each dominium. Pain during intercourse was not correlated with GAD and PHQ scores in this sample. Desire presented a negative correlation with questions related to anhedonia, tiredness, suicidal thoughts, getting easily upset and the feeling that something terrible will happen. Total score of arousal, lubrication, orgasm and satisfaction also presented a significant negative moderate correlation with most of GAD-7 and PHQ-9 scales.

Burnout was also assessed by the total score of each domain and a significant positive correlation was observed with scores of depersonalization and emotional exhaustion with GAD-7 and PHQ-9.

Between depersonalization and emotional exhaustion was also observed a significant and a negative correlation with arousal, lubrication, orgasm and sexual satisfaction domains in FSFI scale. In contrast, when assessing personal accomplishment, there is a negative correlation with GAD-7 and PHQ-9, and a positive correlation with desire, arousal, lubrication, orgasm and sexual satisfaction.

At start, only covariance of anxiety and depression was included, fitting a reasonable fit (CFI = 0.993, X^2^/df = 1.838, RMSE 90% CI = 0.047 [0.04–0.053], p-value = 0.797 and SRMR = 0.063). In order to improve fit and convergence of the model, based on modification indexes over 10, we added covariances among questions 1 and 2 of PHQ, and first three items of GAD. Covariances between lubrication and orgasm, pain during intercourse and satisfaction, pain during intercourse and desire from FSFI were also added. The final model presented an excellent fit (CFI = 0.995, X^2^/df = 1.56, RMSE 90% CI = 0.038 [0.03–0.045], p-value = 0.997 and SRMR = 0.059).

**Fig 2:**
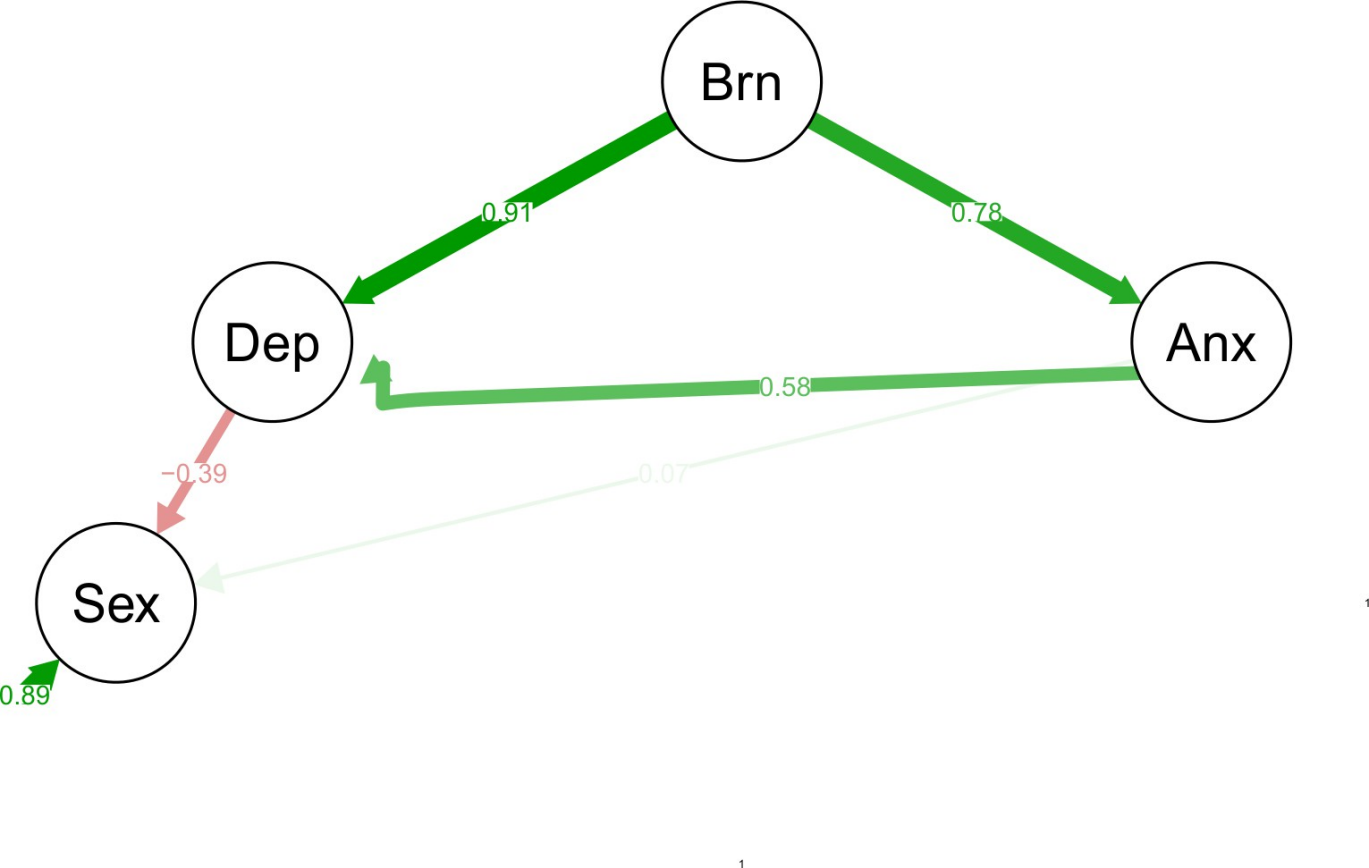
Structural equation model framework. Summarized Plot of SEM Model, variables (anxiety, depression, sexual dysfunction, burnout) are displayed in circles, since they are latent variables, composed by its scores. Green arrows represent positive coefficients and red arrows represent negative coefficients, thickness of arrows is proportional and opacity inversely proportional to relation strength. To improve visualization, scores and covariations were hidden.

In our final model, burnout presented a significant positive effect on depression (**β** = 0,22, p-value < 0.0001) and anxiety (**β** = 0,20, p-value < 0.0001). In contrast, depression (**β** = - 0,07, p-value = 0,013) presented a significant negative effect on sexual function while anxiety a non-significant positive effect on sexual function. Covariance among anxiety and depression has also been significant (**β** = 0,09, p-value = 0,001) (Fig 2).

## Discussion

In this survey, female doctors’ sexual function was mediated for depression and anxiety during COVID-19 pandemic. In our data, we have found a prevalence of sexual dysfunction of 72.7%, higher than the prevalence found among Chinese doctors before the pandemic, which was 49.7% in females [20]. The median FSFI total score was 23.8 in the study population, lower than 28.68 found in the general population studied in Italy (6) and 25.8 in women in childbearing age from Poland [21], possibly because our study addresses a specific population.

Noteworthy, 49.2% of this population achieved criteria for depression by PHQ-9, commonly used to screening participants [22], which is comparable to the 56,3% of depression found in a study in Libya with doctors and nurses during the civil war and COVID-19 pandemic [23] and 45,5%, between healthcare providers in Taiwan during the beginning of the COVID-19 pandemic [24]. Attracting special attention, from the 52% of the participants who are frontline workers, 61% met criteria for anxiety and/or depression.

Burnout was positively correlated with anxiety and depression, with higher indexes of burnout scale between participants with mental illness. During the lockdown, the work environment became atypical and dealing with new protocols and patients significantly increased the rates of burnout and anxiety [25] while providing adequate training to professionals works as a protective factor [26].

Worldwide, COVID-19 epidemic and the restrictive social distancing measures have negatively influenced the sexual function and quality of life in noninfected reproductive-age women who live with their sexual partners [27]. Healthcare workers’ sexual desires decreased, the number of sexual intercourses decreased, their foreplay times decreased, and their sexual intercourse positions changed to less face to face [28]. Although there is no data about the sexual intercourse frequency in the study population before the pandemic, most of them had a sexual intercourse frequency of once a week, in accordance with the finding of these authors.

Working in the frontline was an independent risk factor to worse mental health outcomes in all dimensions of interest in the study population. It was already reported that causes can vary, but for those in the frontline in particular, a lack of opportunity to adequately rest and sleep is likely related to an extremely large load of work, and a lack of personal protective equipment or training may exacerbate mental health impacts [29].

Our findings have a great importance considering that the COVID-19 pandemic has not ended yet. Sexuality and mental health are closely related, and this research instigates a concern with the quality of life of health workers.

The data presented must be considered within its limitations. This is a cross-sectional study, aiming to explore the problem in a specific population. There was no sample size calculated and participants were recruited via digital media and social networks, meaning that we were unable to identify respondents and non-respondents, which can lead to a biased sample. Unfortunately, we have no data collected from this population for a before and after comparison. During the COVID-19 pandemic, many professionals left their houses to avoid family contamination, and this was not considered.

In conclusion, a high index of depression and/or anxiety was shown in the studied population, with almost 80% of them reaching criteria for sexual dysfunction. Depression and anxiety were found as potential mediators of burnout effect on sexual function. However, it is difficult to dissociate the effect of social restriction measures, longer working days, since these factors may be related both to psychiatric diseases and sexual dysfunction. Even so, promoting a lighter work and home environment can be the first step in the promotion of sexual satisfaction and a decrease in depression and anxiety rates during the COVID-19 pandemic for female doctors.

Having found a high prevalence of sexual dysfunction and mental illness reinforces the importance of reassessing this population after the pandemic. As doctors are professionals who deal with the population, their sexuality and mental health must be a concern. It is of great importance to take care of their sexual health, improving their quality of life and global satisfaction.

## Supporting information

S1 Table Linear multivariable model. Models described on Table 3, with coefficients and 95% CI of all variables displayed in columns. Variables added to the model in each row.
(DOCX)

S2 Table Regression coefficients, 95% CI output of structural equation models. (DOCX)

S1 Data (XLSX)

S1 File (DOCX)
