# Supplementary material for: Impact of the covid-19 pandemic on mental health and sexuality of female doctors
Source: PLoS One. 2023 Jul 10;18(7):e0281321. doi: 10.1371/journal.pone.0281321 (PMC10332575; doi:10.1371/journal.pone.0281321)
Supplement: S1 Table — Models described on Table 3, with coefficients and 95% CI of all variables displayed in columns. Variables added to the model in each row. (DOCX) [file pone.0281321.s001.docx]

| Dependent Variable |  | Independent  Variable | Estimate | S.E | z | p-value | lower CI | upper CI |
| --- | --- | --- | --- | --- | --- | --- | --- | --- |
| Sex | =~ | Sexdor | 1.00 | 0.00 | NA | NA | 1.00 | 1.00 |
| Sex | =~ | Sexsatisf | 9.40 | 1.97 | 4.78 | 0.00 | 5.55 | 13.25 |
| Sex | =~ | Sexorgas | 8.76 | 1.73 | 5.07 | 0.00 | 5.37 | 12.15 |
| Sex | =~ | Sexlub | 8.15 | 1.65 | 4.93 | 0.00 | 4.91 | 11.39 |
| Sex | =~ | Sexdesejo | 4.89 | 1.19 | 4.10 | 0.00 | 2.56 | 7.23 |
| Sex | =~ | Sexexcitacao | 8.70 | 1.74 | 5.00 | 0.00 | 5.28 | 12.11 |
| Burn | =~ | BURDesp | 1.00 | 0.00 | NA | NA | 1.00 | 1.00 |
| Burn | =~ | BURnRP | -1.04 | 0.13 | -8.35 | 0.00 | -1.29 | -0.80 |
| Burn | =~ | BUREE | 2.77 | 0.30 | 9.14 | 0.00 | 2.17 | 3.36 |
| Dep | =~ | PHQ1 | 1.00 | 0.00 | NA | NA | 1.00 | 1.00 |
| Dep | =~ | PHQ2 | 1.08 | 0.03 | 34.51 | 0.00 | 1.02 | 1.15 |
| Dep | =~ | PHQ3 | 0.77 | 0.05 | 16.73 | 0.00 | 0.68 | 0.86 |
| Dep | =~ | PHQ4 | 1.07 | 0.04 | 29.31 | 0.00 | 1.00 | 1.15 |
| Dep | =~ | PHQ6 | 0.96 | 0.04 | 27.28 | 0.00 | 0.89 | 1.03 |
| Dep | =~ | PHQ7 | 0.93 | 0.04 | 25.70 | 0.00 | 0.86 | 1.00 |
| Dep | =~ | PHQ8 | 0.94 | 0.04 | 22.09 | 0.00 | 0.86 | 1.03 |
| Dep | =~ | PHQ9 | 0.77 | 0.08 | 9.06 | 0.00 | 0.60 | 0.93 |
| Anx | =~ | GAD1 | 1.00 | 0.00 | NA | NA | 1.00 | 1.00 |
| Anx | =~ | GAD2 | 0.91 | 0.03 | 29.22 | 0.00 | 0.85 | 0.97 |
| Anx | =~ | GAD3 | 1.00 | 0.03 | 38.80 | 0.00 | 0.95 | 1.05 |
| Anx | =~ | GAD4 | 1.05 | 0.03 | 39.69 | 0.00 | 1.00 | 1.10 |
| Anx | =~ | GAD5 | 0.86 | 0.04 | 21.32 | 0.00 | 0.78 | 0.94 |
| Anx | =~ | GAD6 | 0.97 | 0.03 | 36.38 | 0.00 | 0.92 | 1.02 |
| Anx | =~ | GAD7 | 0.78 | 0.04 | 18.50 | 0.00 | 0.70 | 0.86 |
| Sex | ~ | Dep | -0.08 | 0.03 | -2.85 | 0.00 | -0.13 | -0.02 |
| Sex | ~ | Anx | 0.01 | 0.02 | 0.40 | 0.69 | -0.03 | 0.05 |
| Dep | ~ | Burn | 0.23 | 0.02 | 9.91 | 0.00 | 0.19 | 0.28 |
| Anx | ~ | Burn | 0.21 | 0.02 | 9.77 | 0.00 | 0.17 | 0.25 |
| Dep | ~~ | Anx | 0.10 | 0.03 | 3.13 | 0.00 | 0.04 | 0.17 |
| PHQ1 | \| | t1 | -0.94 | 0.08 | -12.55 | 0.00 | -1.09 | -0.80 |
| PHQ1 | \| | t2 | 0.48 | 0.07 | 7.27 | 0.00 | 0.35 | 0.61 |
| PHQ1 | \| | t3 | 1.02 | 0.08 | 13.16 | 0.00 | 0.87 | 1.17 |
| PHQ2 | \| | t1 | -0.78 | 0.07 | -10.89 | 0.00 | -0.91 | -0.64 |
| PHQ2 | \| | t2 | 0.50 | 0.07 | 7.47 | 0.00 | 0.37 | 0.63 |
| PHQ2 | \| | t3 | 1.22 | 0.08 | 14.48 | 0.00 | 1.06 | 1.39 |
| PHQ3 | \| | t1 | -0.95 | 0.08 | -12.64 | 0.00 | -1.10 | -0.81 |
| PHQ3 | \| | t2 | 0.02 | 0.06 | 0.30 | 0.76 | -0.11 | 0.14 |
| PHQ3 | \| | t3 | 0.61 | 0.07 | 8.95 | 0.00 | 0.48 | 0.74 |
| PHQ4 | \| | t1 | -1.65 | 0.11 | -15.30 | 0.00 | -1.87 | -1.44 |
| PHQ4 | \| | t2 | -0.25 | 0.06 | -3.85 | 0.00 | -0.37 | -0.12 |
| PHQ4 | \| | t3 | 0.40 | 0.07 | 6.07 | 0.00 | 0.27 | 0.53 |
| PHQ6 | \| | t1 | -0.30 | 0.06 | -4.66 | 0.00 | -0.43 | -0.17 |
| PHQ6 | \| | t2 | 0.49 | 0.07 | 7.37 | 0.00 | 0.36 | 0.62 |
| PHQ6 | \| | t3 | 1.17 | 0.08 | 14.20 | 0.00 | 1.01 | 1.33 |
| PHQ7 | \| | t1 | -0.87 | 0.07 | -11.83 | 0.00 | -1.01 | -0.72 |
| PHQ7 | \| | t2 | 0.18 | 0.06 | 2.84 | 0.00 | 0.06 | 0.31 |
| PHQ7 | \| | t3 | 0.94 | 0.08 | 12.55 | 0.00 | 0.80 | 1.09 |
| PHQ8 | \| | t1 | 0.08 | 0.06 | 1.22 | 0.22 | -0.05 | 0.20 |
| PHQ8 | \| | t2 | 0.83 | 0.07 | 11.46 | 0.00 | 0.69 | 0.97 |
| PHQ8 | \| | t3 | 1.46 | 0.10 | 15.25 | 0.00 | 1.27 | 1.65 |
| PHQ9 | \| | t1 | 1.42 | 0.09 | 15.19 | 0.00 | 1.24 | 1.61 |
| PHQ9 | \| | t2 | 2.16 | 0.16 | 13.38 | 0.00 | 1.84 | 2.47 |
| PHQ9 | \| | t3 | 2.80 | 0.32 | 8.66 | 0.00 | 2.16 | 3.43 |
| GAD1 | \| | t1 | -1.29 | 0.09 | -14.80 | 0.00 | -1.46 | -1.12 |
| GAD1 | \| | t2 | -0.15 | 0.06 | -2.33 | 0.02 | -0.27 | -0.02 |
| GAD1 | \| | t3 | 0.69 | 0.07 | 9.93 | 0.00 | 0.55 | 0.83 |
| GAD2 | \| | t1 | -0.82 | 0.07 | -11.36 | 0.00 | -0.96 | -0.68 |
| GAD2 | \| | t2 | 0.25 | 0.06 | 3.85 | 0.00 | 0.12 | 0.37 |
| GAD2 | \| | t3 | 1.01 | 0.08 | 13.08 | 0.00 | 0.86 | 1.16 |
| GAD3 | \| | t1 | -1.41 | 0.09 | -15.15 | 0.00 | -1.59 | -1.22 |
| GAD3 | \| | t2 | -0.36 | 0.07 | -5.56 | 0.00 | -0.49 | -0.24 |
| GAD3 | \| | t3 | 0.49 | 0.07 | 7.37 | 0.00 | 0.36 | 0.62 |
| GAD4 | \| | t1 | -1.14 | 0.08 | -14.05 | 0.00 | -1.30 | -0.98 |
| GAD4 | \| | t2 | -0.12 | 0.06 | -1.93 | 0.05 | -0.25 | 0.00 |
| GAD4 | \| | t3 | 0.61 | 0.07 | 8.95 | 0.00 | 0.48 | 0.74 |
| GAD5 | \| | t1 | 0.09 | 0.06 | 1.42 | 0.16 | -0.03 | 0.22 |
| GAD5 | \| | t2 | 0.92 | 0.07 | 12.37 | 0.00 | 0.78 | 1.07 |
| GAD5 | \| | t3 | 1.87 | 0.13 | 14.81 | 0.00 | 1.62 | 2.11 |
| GAD6 | \| | t1 | -1.18 | 0.08 | -14.27 | 0.00 | -1.34 | -1.02 |
| GAD6 | \| | t2 | 0.05 | 0.06 | 0.71 | 0.48 | -0.08 | 0.17 |
| GAD6 | \| | t3 | 0.91 | 0.07 | 12.28 | 0.00 | 0.77 | 1.06 |
| GAD7 | \| | t1 | -0.18 | 0.06 | -2.84 | 0.00 | -0.31 | -0.06 |
| GAD7 | \| | t2 | 0.67 | 0.07 | 9.64 | 0.00 | 0.53 | 0.80 |
| GAD7 | \| | t3 | 1.32 | 0.09 | 14.91 | 0.00 | 1.15 | 1.50 |
| Sexdor | ~~ | Sexdor | 1.22 | 0.08 | 15.90 | 0.00 | 1.07 | 1.37 |
| Sexsatisf | ~~ | Sexsatisf | 0.37 | 0.26 | 1.44 | 0.15 | -0.14 | 0.89 |
| Sexorgas | ~~ | Sexorgas | 0.73 | 0.17 | 4.26 | 0.00 | 0.40 | 1.07 |
| Sexlub | ~~ | Sexlub | 0.94 | 0.18 | 5.32 | 0.00 | 0.59 | 1.28 |
| Sexdesejo | ~~ | Sexdesejo | 3.97 | 0.40 | 9.82 | 0.00 | 3.18 | 4.77 |
| Sexexcitacao | ~~ | Sexexcitacao | 0.37 | 0.13 | 2.91 | 0.00 | 0.12 | 0.61 |
| BURDesp | ~~ | BURDesp | 20.38 | 1.52 | 13.42 | 0.00 | 17.40 | 23.35 |
| BURnRP | ~~ | BURnRP | 24.07 | 1.88 | 12.79 | 0.00 | 20.38 | 27.76 |
| BUREE | ~~ | BUREE | 44.37 | 4.92 | 9.02 | 0.00 | 34.73 | 54.02 |
| PHQ1 | ~~ | PHQ1 | 0.34 | 0.00 | NA | NA | 0.34 | 0.34 |
| PHQ2 | ~~ | PHQ2 | 0.22 | 0.00 | NA | NA | 0.22 | 0.22 |
| PHQ3 | ~~ | PHQ3 | 0.61 | 0.00 | NA | NA | 0.61 | 0.61 |
| PHQ4 | ~~ | PHQ4 | 0.24 | 0.00 | NA | NA | 0.24 | 0.24 |
| PHQ6 | ~~ | PHQ6 | 0.38 | 0.00 | NA | NA | 0.38 | 0.38 |
| PHQ7 | ~~ | PHQ7 | 0.43 | 0.00 | NA | NA | 0.43 | 0.43 |
| PHQ8 | ~~ | PHQ8 | 0.41 | 0.00 | NA | NA | 0.41 | 0.41 |
| PHQ9 | ~~ | PHQ9 | 0.61 | 0.00 | NA | NA | 0.61 | 0.61 |
| GAD1 | ~~ | GAD1 | 0.26 | 0.00 | NA | NA | 0.26 | 0.26 |
| GAD2 | ~~ | GAD2 | 0.39 | 0.00 | NA | NA | 0.39 | 0.39 |
| GAD3 | ~~ | GAD3 | 0.26 | 0.00 | NA | NA | 0.26 | 0.26 |
| GAD4 | ~~ | GAD4 | 0.18 | 0.00 | NA | NA | 0.18 | 0.18 |
| GAD5 | ~~ | GAD5 | 0.45 | 0.00 | NA | NA | 0.45 | 0.45 |
| GAD6 | ~~ | GAD6 | 0.30 | 0.00 | NA | NA | 0.30 | 0.30 |
| GAD7 | ~~ | GAD7 | 0.55 | 0.00 | NA | NA | 0.55 | 0.55 |
| Sex | ~~ | Sex | 0.03 | 0.01 | 2.70 | 0.01 | 0.01 | 0.05 |
| Burn | ~~ | Burn | 9.93 | 1.90 | 5.23 | 0.00 | 6.21 | 13.64 |
| Dep | ~~ | Dep | 0.13 | 0.04 | 3.66 | 0.00 | 0.06 | 0.20 |
| Anx | ~~ | Anx | 0.31 | 0.04 | 7.83 | 0.00 | 0.23 | 0.39 |
| PHQ1 | ~*~ | PHQ1 | 1.00 | 0.00 | NA | NA | 1.00 | 1.00 |
| PHQ2 | ~*~ | PHQ2 | 1.00 | 0.00 | NA | NA | 1.00 | 1.00 |
| PHQ3 | ~*~ | PHQ3 | 1.00 | 0.00 | NA | NA | 1.00 | 1.00 |
| PHQ4 | ~*~ | PHQ4 | 1.00 | 0.00 | NA | NA | 1.00 | 1.00 |
| PHQ6 | ~*~ | PHQ6 | 1.00 | 0.00 | NA | NA | 1.00 | 1.00 |
| PHQ7 | ~*~ | PHQ7 | 1.00 | 0.00 | NA | NA | 1.00 | 1.00 |
| PHQ8 | ~*~ | PHQ8 | 1.00 | 0.00 | NA | NA | 1.00 | 1.00 |
| PHQ9 | ~*~ | PHQ9 | 1.00 | 0.00 | NA | NA | 1.00 | 1.00 |
| GAD1 | ~*~ | GAD1 | 1.00 | 0.00 | NA | NA | 1.00 | 1.00 |
| GAD2 | ~*~ | GAD2 | 1.00 | 0.00 | NA | NA | 1.00 | 1.00 |
| GAD3 | ~*~ | GAD3 | 1.00 | 0.00 | NA | NA | 1.00 | 1.00 |
| GAD4 | ~*~ | GAD4 | 1.00 | 0.00 | NA | NA | 1.00 | 1.00 |
| GAD5 | ~*~ | GAD5 | 1.00 | 0.00 | NA | NA | 1.00 | 1.00 |
| GAD6 | ~*~ | GAD6 | 1.00 | 0.00 | NA | NA | 1.00 | 1.00 |
| GAD7 | ~*~ | GAD7 | 1.00 | 0.00 | NA | NA | 1.00 | 1.00 |
