# Supplementary material for: Impact of the covid-19 pandemic on mental health and sexuality of female doctors
Source: PLoS One. 2023 Jul 10;18(7):e0281321. doi: 10.1371/journal.pone.0281321 (PMC10332575; doi:10.1371/journal.pone.0281321)
Supplement: S2 Table — (DOCX) [file pone.0281321.s002.docx]

|  | *Dependent variable:* | | | | | | |
| --- | --- | --- | --- | --- | --- | --- | --- |
|  | (1) | (2) | (3) | (4) | (5) | (6) | (7) |
| Mental illness | -3.09^***^(-4.45, -1.73) | -3.53^***^(-4.88, -2.18) | -2.83^***^ (-3.98, -1.68) | -2.61^***^(-3.77, -1.46) | -2.65^***^  (-3.80, -1.50) | -2.55^***^  (-3.70, -1.41) | -2.47^***^ (-3.64, -1.30) |
| Agecat> =40yr |  | -1.70^**^ (-3.24, -0.17) | -0.84  (-2.14, 0.47) | -1.13^*^  (-2.46, 0.19) | -1.10  (-2.43, 0.23) | -1.30^*^  (-2.58, -0.01)^*^ | -1.43^**^  (-2.75, -0.10) |
| Sexual intercourse more than 4 times a week - Yes |  |  | 10.82^***^(8.49, 13.15) | 10.94^***^ (8.66, 13.21) | 11.03^***^  (8.77, 13.28) | 10.17^***^  (7.62, 12.72) | 10.16^***^  (7.60, 12.72) |
| Physical exercise more than 150 minutes/week |  |  |  | 1.60^**^ (0.38, 2.83) | 1.44^**^  (0.22, 2.65) | 1.48^**^  (0.27, 2.70) | 1.46^**^  (0.25, 2.68) |
| Sexual orientation  Bisexual |  |  |  |  | 1.98 (-1.88, 5.83) | 1.85 (-2.25, 5.95) | 1.81  (-2.37, 5.98) |
| Sexual orientation Homosexual |  |  |  |  | -3.43^**^ (-6.65, -0.22) | -3.44^**^  (-6.53, -0.36) | -3.48^**^  (-6.60, -0.37) |
| Steady partner |  |  |  |  |  | 1.78 (-0.55, 4.10) | 1.73  (-0.60, 4.07) |
| Menstrual cycle - irregular during the pandemic |  |  |  |  |  |  | -1.06  (-3.23, 1.11) |
| Menstrual cycle - do not menstruate |  |  |  |  |  |  | 0.29  (-0.94, 1.51) |
| Constant | 23.63^***^ (22.68, 24.58) | 24.42^***^ (23.38, 25.46) | 14.79^***^ (12.34, 17.23) | 14.02^***^ (11.62, 16.42) | 14.06^***^(11.68, 16.44) | 13.26^***^  (10.69, 15.84 | 13.30^***^  (10.70, 15.90) |
| *Note:* | ^*^p<0.1; ^**^p<0.05; ^***^p<0.01 | | | | | | |

| ***Dependent variable (FSFI score)*** | | | | | |  |
| --- | --- | --- | --- | --- | --- | --- |
| **Independent Variable**  **Psychiatric Disease** | **𝛃 (95% CI)** | **t value** | **p-value** | **logLik** | **AIC** | **BIC** |
| (1) | -3.09^***^(-4.45, -1.73) | -4,4428 | 0,00001161 | -1305,50 | 2616,99 | 2628,88 |
| (2) | -3.53^***^(-4.88, -2.18) | -5,1309 | 4,582E-07 | -1303,20 | 2614,41 | 2630,25 |
| (3) | -2.83^***^ (-3.98, -1.68) | -4,8371 | 1,911E-06 | -1222,21 | 2454,43 | 2474,23 |
| (4) | -2.61^***^(-3.77, -1.46) | -4,4463 | 0,00001146 | -1218,68 | 2449,36 | 2473,13 |
| (5) | -2.65^***^ (-3.80, -1.50) | -4,5055 | 8,818E-06 | -1215,64 | 2447,29 | 2478,98 |
| (6) | -2.55^***^ (-3.70, -1.41) | -4,363 | 0,00001655 | -1213,68 | 2445,36 | 2481,01 |
| (7) | -2.47^***^ (-3.64, -1.30) | -4,1304 | 0,00004461 | -1212,84 | 2447,67 | 2491,24 |
