# Supplementary material for: Impact of the covid-19 pandemic on mental health and sexuality of female doctors
Source: PLoS One. 2023 Jul 10;18(7):e0281321. doi: 10.1371/journal.pone.0281321 (PMC10332575; doi:10.1371/journal.pone.0281321)
Supplement: S1 File — (DOCX) [file pone.0281321.s004.docx]

Data_dictionary

Ziegler N

null

## Warning: package 'flextable' was built under R version 4.1.1

##
## Attaching package: 'flextable'

## The following object is masked from 'package:purrr':
##
## compose

| **variable_name** | **variable_class** | **variable_description** | **variable_options** |
| --- | --- | --- | --- |
| ID | character | Patient ID | 1 to 99 |
| Psychiatric_Disease |  | Any Psychiatric Disease, defined either by PHQ-9/GAD-7/Maslach scores | Y |
|  |  |  | N |
| Idade | numeric | Age in years (self reported) | 23 to 76 |
| BMI |  | BMI (calculated from self reported weight and height | 17.3010380622837 to 37.8138020377438 |
| Steady_Partner |  | Steady partner | N |
|  |  |  | Y |
| Menstrual_Cycle |  | Question about changes on the regularity of Menstrual Cycle | Keep the same pattern |
|  |  |  | Do not menstruate |
|  |  |  | Became Irregular |
| Living |  | Test | Alone |
|  |  |  | Partner |
|  |  |  | Partner and Others |
|  |  |  | Others |
| Regular_Physical_activity |  | Self reported regular physical activity (>150 minutes/week) | N |
|  |  |  | Y |
| Alcohol_consumption |  | Alcohol comsumption in last four weeks | 2-4 per month |
|  |  |  | 1 per month |
|  |  |  | Never |
|  |  |  | 2-3 per wk |
|  |  |  | 3+ per wk |
| Sex_relation_4wks |  |  | N |
|  |  |  | Y |
| Frequency_sex_relation |  | Self reported frequency of sex relations in last four weeks | 1 per month |
|  |  |  | 3+ per wk |
|  |  |  | 2-3 per wk |
|  |  |  | 1-2 per month |
|  |  |  | 1 per wk |
| FSFI_desire |  | Sum of the scores of questions correspondent to desire dominium of FSFI | 2 to 10 |
| FSFI_excitation |  | Sum of the scores of questions correspondent to excitation dominium of FSFI | 0 to 6 |
| FSFI_lubrication |  | Sum of the scores of questions correspondent to lubrication dominium of FSFI | 0 to 6 |
| FSFI_orgasm |  | Sum of the scores of questions correspondent to orgasm dominium of FSFI | 0 to 6 |
| FSFI_satisfaction |  | Sum of the scores of questions correspondent to satisfaction dominium of FSFI | 0.4 to 6 |
| FSFI_pain |  | Sum of the scores of questions correspondent to pain dominium of FSFI | 0 to 6 |
| scoreFSFIfinal |  | Total FSFI score (sum of the scores, weighted by dominium) | 2.2 to 30.7 |
| FSFI1 |  | Item 1 of FSFI | 1 to 5 |
| FSFI2 |  | Item 2 of FSFI | 1 to 5 |
| FSFI3 |  | Item 3 of FSFI | 0 to 5 |
| FSFI4 |  | Item 4 of FSFI | 0 to 5 |
| FSFI5 |  | Item 5 of FSFI | 0 to 5 |
| FSFI6 |  | Item 6 of FSFI | 0 to 5 |
| FSFI7 |  | Item 7 of FSFI | 0 to 5 |
| FSFI8 |  | Item 8 of FSFI | 0 to 5 |
| FSFI9 |  | Item 9 of FSFI | 0 to 5 |
| FSFI10 |  | Item 10 of FSFI | 0 to 5 |
| FSFI11 |  | Item 11 of FSFI | 0 to 5 |
| FSFI12 |  | Item 12 of FSFI | 0 to 5 |
| FSFI13 |  | Item 13 of FSFI | 0 to 5 |
| FSFI14 |  | Item 14 of FSFI | 0 to 5 |
| FSFI15 |  | Item 15 of FSFI | 0 to 5 |
| FSFI16 |  | Item 16 of FSFI | 1 to 5 |
| FSFI17 |  | Item 17 of FSFI | 0 to 5 |
| FSFI18 |  | Item 18 of FSFI | 0 to 5 |
| FSFI19 |  | Item 19 of FSFI | 0 to 5 |
| BUR1 |  | Item 1 of Maslach Burnout Scale | 0 to 6 |
| BUR2 |  | Item 2 of Maslach Burnout Scale | 0 to 6 |
| BUR3 |  | Item 3 of Maslach Burnout Scale | 0 to 6 |
| BUR4 |  | Item 4 of Maslach Burnout Scale | 1 to 6 |
| BUR5 |  | Item 5 of Maslach Burnout Scale | 0 to 6 |
| BUR6 |  | Item 6 of Maslach Burnout Scale | 0 to 6 |
| BUR7 |  | Item 7 of Maslach Burnout Scale | 0 to 6 |
| BUR8 |  | Item 8 of Maslach Burnout Scale | 0 to 6 |
| BUR9 |  | Item 9 of Maslach Burnout Scale | 0 to 6 |
| BUR10 |  | Item 10 of Maslach Burnout Scale | 0 to 6 |
| BUR11 |  | Item 11 of Maslach Burnout Scale | 0 to 6 |
| BUR12 |  | Item 12 of Maslach Burnout Scale | 0 to 6 |
| BUR13 |  | Item 13 of Maslach Burnout Scale | 0 to 6 |
| BUR14 |  | Item 14 of Maslach Burnout Scale | 0 to 6 |
| BUR15 |  | Item 15 of Maslach Burnout Scale | 0 to 6 |
| BUR16 |  | Item 16 of Maslach Burnout Scale | 0 to 6 |
| BUR17 |  | Item 17 of Maslach Burnout Scale | 1 to 6 |
| BUR18 |  | Item 18 of Maslach Burnout Scale | 0 to 6 |
| BUR19 |  | Item 19 of Maslach Burnout Scale | 0 to 6 |
| BUR20 |  | Item 20 of Maslach Burnout Scale | 0 to 6 |
| BUR21 |  | Item 21 of Maslach Burnout Scale | 0 to 6 |
| BUR22 |  | Item 22 of Maslach Burnout Scale | 0 to 6 |
| PHQ1 |  | Item 1 of PHQ-9 Scale | 0 to 3 |
| PHQ2 |  | Item 2 of PHQ-9 Scale | 0 to 3 |
| PHQ3 |  | Item 3 of PHQ-9 Scale | 0 to 3 |
| PHQ4 |  | Item 4 of PHQ-9 Scale | 0 to 3 |
| PHQ5 |  | Item 5 of PHQ-9 Scale | 0 to 3 |
| PHQ6 |  | Item 6 of PHQ-9 Scale | 0 to 3 |
| PHQ7 |  | Item 7 of PHQ-9 Scale | 0 to 3 |
| PHQ8 |  | Item 8 of PHQ-9 Scale | 0 to 3 |
| PHQ9 |  | Item 9 of PHQ-9 Scale | 0 to 3 |
| GAD1 |  | Item 1 of GAD-7 Scale | 0 to 3 |
| GAD2 |  | Item 2 of GAD-7 Scale | 0 to 3 |
| GAD3 |  | Item 3 of GAD-7 Scale | 0 to 3 |
| GAD4 |  | Item 4 of GAD-7 Scale | 0 to 3 |
| GAD5 |  | Item 5 of GAD-7 Scale | 0 to 3 |
| GAD6 |  | Item 6 of GAD-7 Scale | 0 to 3 |
| GAD7 |  | Item 7 of GAD-7 Scale | 0 to 3 |
| Maslach_Emotional_exhaustion |  | Sum of scores of items related to emotional exhaustion from Maslach Burnout Scale | 1 to 48 |
| Maslach_PR |  | Sum of scores of items related to professional realization from Maslach Burnout Scale | 18 to 48 |
| Maslach_Despair |  | Sum of scores of items related to despair from Maslach Burnout Scale | 0 to 27 |
| PHQ9_total |  | Sum of scores of items from PHQ-9 scale | 0 to 23 |
| GAD7_total |  | Sum of scores of items from GAD-7 scale | 0 to 21 |
| Maslach_cathegorical |  | Dichotomized Maslach Burnout Scale | N |
|  |  |  | Y |
| PHQ_Cathegorical |  | Dichotomized PHQ-9 (>9) Scale | Y |
|  |  |  | N |
| GAD_Cathegorical |  | Dichotomized GAD-7 (>7) Scale | Y |
|  |  |  | N |
| Sex_dysfunction |  | Dichotomized FSFI (<26.55) Scale | Y |
|  |  |  | N |
